# Upper Limits of Normal for Serum Alanine Aminotransferase Levels in Chinese Han Population

**DOI:** 10.1371/journal.pone.0043736

**Published:** 2012-09-04

**Authors:** Ming-Hua Zheng, Ke-Qing Shi, Yu-Chen Fan, Wen-Yue Liu, Xian-Feng Lin, Ling-Fei Li, Yong-Ping Chen

**Affiliations:** 1 Department of Infection and Liver Diseases, Liver Research Center, the First Affiliated Hospital of Wenzhou Medical College, Wenzhou, China; 2 Institute of Hepatology, Wenzhou Medical College, Wenzhou, China; 3 Department of Hepatology, Qilu Hospital of Shandong University, Jinan, China; 4 School of the First Clinical Medical Sciences, Wenzhou Medical College, Wenzhou, China; The University of Hong Kong, Hong Kong

## Abstract

**Background and Objectives:**

Serum alanine aminotransferase (ALT) activity is the most common tool for the assessment of liver diseases. However, it is not clear whether the current normal ALT range really discriminate patients with or without liver diseases. The present study was to establish a new normal range of ALT and examine its ability to identify patients with hepatitis B or nonalcoholic fatty liver disease (NAFLD) in Chinese Han population.

**Methods:**

53037 adults were included in this study from January 1st 2008 to August 31st 2010. The 95th percentile of ALT in population with relative low risk factors for liver diseases was set as the new upper limits of normal ALT in gender-specific manner.

**Results:**

The 95^th^ percentile levels at low risk factors for liver diseases were achieved at 35 U/L for men and 23 U/L for women. The concordance statistics for detection were 0.873 (95%CI: 0.865–0.881) for HBV and 0.932 (95%CI: 0.927–0.937) for NAFLD in men while 0.857 (95%CI: 0.850–0.864) for HBV and 0.909 (95%CI: 0.903–0.915) for NAFLD in women. The median sensitivity of the current used ALT upper limit (40 U/L) was 6.6% for HBV and 29.7% for NAFLD and median specificity was 98.7% for men and 99.4% for women. Using our new-derived thresholds, the sensitivities ranged from 35.3% to 61.1% and the specificities were 94.8% for men and 94.6% for women.

**Conclusions:**

Our results suggest that upper limits of ALT 35 U/L for men and 23 U/L for women in Chinese Han population. Re-consideration of normal limits of ALT should be recommended.

**Trial Registration:**

ChiCTR.org ChiCTR-OCS-11001173

## Introduction

Serum alanine aminotransferase (ALT) concentration is the most widely used tool for the assessment of liver diseases. Upper limit of normal (ULN) ALT is usually the reliable candidate to discriminate normal or abnormal for liver function. Particularly, in the treatment of viral hepatitis, ALT is always used for screening the subjects who need anti-viral therapy [1], [2], [3]. However, it is still not well demonstrated whether the current used ULN standard of ALT is appropriate for clinical events.

Current ULN for ALT level were set, on average, ranging from 30 U/L to 50 U/L over the past 10 years. Such thresholds, however, vary tremendously among hospitals, research centers and geographic locations. Currently, the upper limit of normal ALT has been re-evaluated in different countries by involving different age groups. These studies suggested that the upper limit of normal ALT should be revised and the recommendations are 30 U/L for men and 19 U/L for women respectively [4], [5], [6], [7].

In China, the upper limit of normal ALT was established in the 1950s and has not changed in the past four decades [8]. Since Chinese population has a high background of hepatitis B surface antigen (HBsAg) prevalence [9], [10], and at the same time there are dramatic increases in nonalcoholic fatty liver diseases (NAFLD) due to rapid change in lifestyle of Chinese [1], [11], it is urgent to establish the upper limit of normal ALT for adult population in China. In the present study, we established a new normal range of serum ALT and examined its ability to identify patients with hepatitis B and nonalcoholic fatty liver disease (NAFLD) and those at low risks for liver diseases in Chinese Han population.

## Materials and Methods

### Study population

A prospective cross-sectional study was conducted among healthy examination participants at the First Affiliated Hospital of Wenzhou Medical College from January 1st 2008 to August 31st 2010. A total of 53037 eligible participants (28624 in male and 24413 in female) were included. The ages of participants were from 19 to 44 years old with a mean age of 35.3 (95% confidence interval (CI): 35.1 to 35.3) years old for male and 34.9 (95% CI: 34.8 to 35.0) years old for female, respectively. Informed consent file was written from each participant and the research protocol of the study was approved by the Ethics Committee of the First Affiliated Hospital of Wenzhou Medical College.

The Health Information System was developed by the First Affiliated Hospital of Wenzhou Medical College to collect the health examination data such as age, gender, medication history, health habit, body index, blood pressure, blood test data and hepatic ultrasonic finding. All the examinations were performed in the morning and participants were instructed to refrain from exercise during the day prior to their examinations and fasting overnight before the examination.

Blood pressure was measured using an automated sphygmomanometer with the participants in a sitting position. Systolic blood pressure (SBP) and diastolic blood pressure (DBP) were measured at the first and fifth Korotkoff sounds, respectively. Standing height and body weight without shoes or outer clothes were measured. Body mass index (BMI) was calculated as weight in kilograms divided by height in squared meters.

Blood samples were collected from an antecubital vein and were centrifuged within 30 minutes after collection. All blood tests were performed in the Laboratory of Biochemistry and the Laboratory of Virology at the First Affiliated Hospital of Wenzhou Medical College. The experimental procedures were consistent throughout the study period and the laboratory were certified according to International Organization Standardization. Serum levels of ALT, total cholesterol (TC), triglyceride (TG), high-density lipoprotein cholesterol (HDL-C), low-density lipoprotein cholesterol (LDL-C), uric acid, and fasting plasma glucose (FPG), were determined by the Hitachi 7600 automatic analyzer (Hitachi, Japan). The laboratory intra-assay coefficient of variation (CV) for ALT was 1.2%, and the inter-assay CV over a 2 week period was 2.3%. HBsAg was examined using the Auszyme Monoclonal Test kit (Abbott, AXSYM). Hepatitis C virus (HCV) antibody was tested using the Direct Solid-phase Enzyme Immunoassay kit and human immunodeficiency virus (HIV) antibody was measured using the Synthetic Peptide Enzyme Immunoassay kit, respectively (Tecan Schweiz Rreedom EVOLYZER 150, Switzerland).

Hepatic ultrasonograph was performed in all participants by twenty six experienced sonographers, who were blinded to the clinical and laboratory data throughout the study and had at least 3 years' experience in abdominal ultrasonography at the First Affiliated Hospital of Wenzhou Medical College. The hepatic ultrasonography was obtained by employing the Siemens Acuson Sequoia 512 ultrasound machine (Siemens, Germany) with a 4.0-MHz probe. All ultrasonographic images were stored in an Image-storage Server. Two experienced hepatologists reviewed the images and made the diagnosis of hepatic steatosis without reference to any individual information of the participants. Hepatic steatosis was diagnosed by characteristic echo patterns according to conventional criteria, such as the evidence of diffuse hyperechogenicity of the liver relative to the kidneys, ultrasound beam attenuation, and poor visualization of intrahepatic structures [12]. The concordance statistics of diagnosis made by the two hepatologists was 0.95 (95% CI: 0.93 to 0.97).

### Exclusion and inclusion criteria

In order to define the group of population either with low risk factors for liver disease or without known risk factors, exclusion and inclusion criteria was established. Alcohol consumption was identified according to self-reporting. We excluded participants who drink alcohol greater than 40gram per day for men and 20gram per day for women [1]. Medication history of all participants was reviewed. There were 168 medications and 50 herbs that are known to be associated with hepatotoxicity and/or elevation of ALT (Table 1 and Table 2). Participants who used any of these 218 medications were excluded. Since patients with fatty liver are usually associated with overweight, participants with overweight were excluded and the obesity was defined according to diagnostic standard for Asian population, which is BMI >25 kg/m^2^ for both genders [13].

**Table 1:** List of 168 potentially hepatotoxic medications.

Acarbose
Actargil
Acyclovir
Alanyl glutamine
Albendazole
Allopurinol
Alprazolam
Alprostadil
Ambroxol
Amikacin
Amino acid
Amiodarone
Amitriptyline
Amoxicillin
Amoxicillin/clavulanic
Amoxicillin/sulbactam
Amphotericin B
Analginum
Aripiprazole
Aspirin
Atorvastatin
Azithromycin
Aztreonam
Baclofen
Betahistine
Buspirone
Butylphthalide
Carbamazepine
Carboplatin
Carvedilol
Cefatriaxone
Cefminox
Cefodizime
Cefoperazone
Cefotiam
Cefoxitin
Ceftazidime
Ceftizoxime
Cefuroxime
Celebrex
Cephazolin
Ciclosporin
Cimetidine
Cinepazide maleate
Cisplatin
Citalopram
Clarithromycin
Clindamycin
Clonazepam
Cloxacillin
Clozapine
Colchicin
Cyclophosphamide
Decitabine
Dexibuprofen
Diazepam
Diclofenac
Docetaxel
Donepezil
Dutoxetine
Edaravone
Eperisone
Epirubicin
Erythromycin lactobionate
Escin
Estramustine
Ethambutol
Etimicin
Fasudil
Fenofibrate
Finasteride
Fluconazol
Fluorouracil
Fluoxetine
Fluvastatin
Fusidic
Gastrodin
Gatifloxacin
Gemcitabine
Gestrinone
Glipizide
Gliquidone
Huperzine A
Idarubicin
Imuran
Inderal
Indometacin
Irbesartan
Irinotecan
Isoniazid
Itraconazole
Ketoconazole
Lamivudine
Lansoprazole
Leflunomide
Levofloxacin
Lomefloxacin
Lorazepam
Lornoxicam
Loxoprofen sodiun
Luteosterone
Mannitol
Memantine
Meropenem
Metformin
Methimazole
Methotrexate
Methylprednisolone
Metoprolol
Metronidazole
Mezlocillin
Mifepristone
Mirtazapine
Moxifloxacin
Naloxone
Nateglinide
Nedaplatin
Netilmicin
Nimodipine
Olanzapine
Omeprazole
Ondansetron
Ornidazole
Oxaliplatin
Oxcarbazepine
Oxiracetam
Pantoprazole
Paroxetine
Peflacine
Perindopril
Perphenazine
Phenergan
Phenobarbital
Piracetam
Propafenone
Propylthiouracil
Pseudoephedrine hydrochloride
Pyrazinamide
Quetiapine
Ranitidine
Repaglinide
Reserpine
Rifampicin
Rifapentine
Risperidone
Rosuvastatin calcium
Roxithromycin
Sertraline
Simvastatin
Sodium valproate
Sparfloxacin
Sulfalene
Sulfasalazine
Sulpiride
Tacrolimus
Teicoplanin
Telmisartan
Terbinafine
Tinidazole
Toremifene citrate
Tranexamic acid
Tretinoin
Tropisetron
Vancomycin
Venlafaxine
Vinorelbine
Voriconazole
Ziprasidone

**Table 2:** List of 50 potentially hepatotoxic herbs.

Abrus precatorius
Airpotato yam
Antifeverile dichroa root
Argy wormwood leaf
Atractylodes rhizome
Blister beetle
Calomeas
Camptotheca acuminata
Castor seed
Cattail pollen
Chaulmoogra
Chenopodium ambrosioides
Chinese coriaria leaf
Chinese gall
Climbing groundsel
Clore
Common bletilla tuber
Common rue herb
Cottonseed oil
Cyrtomii rhizoma
Derris
Dioscin
Field grounsel herb
Fleeceflower root
Fourstamen stephania root
Gamboge
Graceful jessamine herb
Gynura root
Hemerocallitis
Herba crotalariae
Java brucea fruit
Lilac daphne flower bud
Lycoris radiata
Medicine terminalia fruit
Natural indigo
Nutmeg
Pomegranate rind
Puncturevine caltrop fruit
Purpleflower holly leaf
Rattlebox
Realgar
Rhubarb
Sanguisorba
Seaweed
Securinine
Siberian cocklebur fruit
Silktree albizia bark
Snakegourd root
Szechwan chinaberry bark
Tripterygium wilfordii

**Figure 1:**
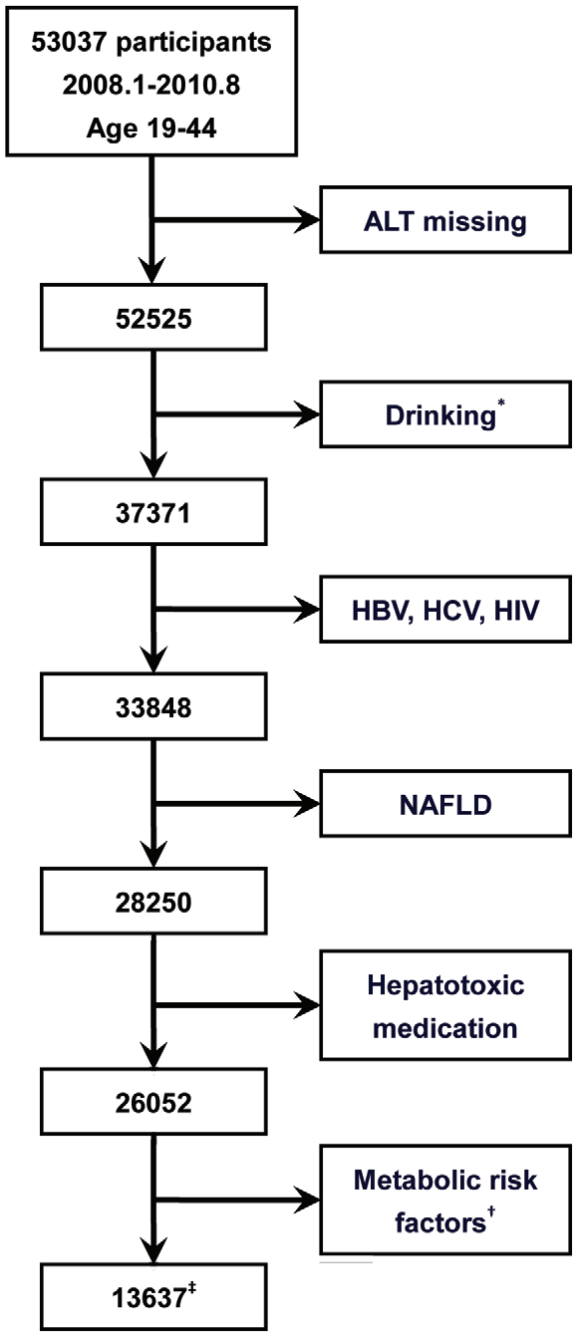
A flow diagram of study participants. ^*^: Alcohol consumption greater than 40 g/d for men and 20 g/d for women. **^†^**: Metabolic risk factors including body mass index >25 kg/m^2^, hypertriglyceridemia (≥1.7 mmol/L), low HDL-C (<1.03 mmol/L for men and <1.29 mmol/L for women), impaired FPG (≥5.6 mmol/L), elevated blood pressure (≥130/85 mmHg), or hyperuricemia (>420 µmol/L for men and >360 µmol/L for women). **^‡^**: Men 4765, women 8872. HBV, hepatitis B virus; HCV, hepatitis C virus; HIV, human immunodeficiency virus; NAFLD, nonalcoholic fatty liver disease.

Because of the association between fatty liver and insulin resistance [14], we excluded those participants who were missing fasting laboratory data, as well as those with any of the following metabolic risk factors for NAFLD: hypertriglyceridemia (≥1.7 mmol/L), low HDL-C (<1.03 mmol/L for men and <1.29 mmol/L for women), impaired FPG (≥5.6 mmol/L), elevated blood pressure (≥130/85 mmHg), or hyperuricemia (>420 µmol/L for men and >360 µmol/L for women) [15], [16]. Table 3 shows the characteristics of fasting laboratory results before and after exclusions. Figure 1 shows the flow of inclusion and exclusion criteria.

**Table 3:** Demographic and metabolic characteristics of the sample before and after exclusions.

	Total sample*		Included sample	
Characteristic	Men (n = 28348)	Women (n = 24177)	Men (n = 4765)	Women (n = 8872)
Mean age (95% CI)–(year)	35.3 (35.1–35.3)	34.9 (34.8–35.0)	33.3 (33.1–33.4)	34.4 (34.2–34.5)
Mean BMI (95% CI)– (kg/m^2^)	23.83 (23.78–23.88)	21.19 (21.14–21.23)	21.13 (21.08–21.19)	20.39 (20.35–20.43)
Mean SBP (95% CI)– (mmHg)	117.4 (117.2–117.6)	106.8 (106.7–107.0)	109.8 (109.7–110.1)	104.1 (103.9–104.3)
Mean DBP (95% CI)–(mmHg)	79.7 (79.5–79.8)	71.6 (71. 5–71.7)	73.4 (73.3–73.6)	69.6 (69.4–69.7)
Mean total cholesterol (95% CI)– (mmol/L)	4.86 (4.84–4.89)	4.50 (4.48–4.52)	4.55 (4.51–4.60)	4.56 (4.53–4.59)
Mean triglyceride (95% CI)– (mmol/L)	1.96 (1.88–2.07)	0.93 (0.91–0.96)	0.96 (0.95–0.97)	0.73 (0.72–0.74)
Mean HDL-C (95% CI)– (mmol/L)	1.25 (1.22–1.29)	1.63 (1.49–1.82)	1.48 (1.39–1.59)	1.89 (1.64–2.23)
Mean LDL-C (95% CI)– (mmol/L)	3.00 (2.98–3.03)	2.62 (2.61–2.64)	2.83 (2.77–2.92)	2.57 (2.55–2.60)
Mean FPG (95% CI)– (mmol/L)	5.38 (5.34–5.40)	5.15 (5.13–5.17)	5.10 (5.08–5.11)	5.04 (5.03–5.05)
Mean uric acid (95% CI)– (µmol/L)	389.1 (387.8–390.6)	269.1 (268.0–270.1)	360.6 (358.3–362.8)	263.8 (262.6–265.1)
Mean ALT (95% CI)– (IU/L)	36.0 (35.5–36.5)	17.4 (17.2–17.7)	16.5 (16.1–16.8)	12.2 (12.0–12.5)

ALT, alanine aminotransferase; BMI, body mass index; SBP, systolic blood pressure; DBP, systolic blood pressure; HDL-C, high-density lipoprotein cholesterol; LDL-C, low-density lipoprotein cholesterol; FPG, fasting plasma glucose; CI, confidence interval.

* : 512 data were missing.

In this present study, we identified normal group as population without any known risk factors for liver disease. The inclusive criteria for normal group was listed as the following: absence of fatty liver using hepatic ultrasonography; no intake of potentially hepatotoxic medications and herbs listed in Table 1 and Table 2; absence of known chronic liver disease (CLD); absence of chronic alcohol consumption; normal laboratory values (biochemical values, HBsAg negative, HCV antibody negative, HIV antibody negative).

### Population groups

Participants were divided into three groups. One was the normal group of participants with normal liver function and the inclusive criteria has been listed as above. The second group was population with chronic hepatitis B and the third group for NAFLD. The clinical and laboratory features of participants in the three groups were listed in Figure S1. Chronic hepatitis B was diagnosed according to persistently positive HBsAg for more than 6 months [2]. NAFLD was diagnosed by abdominal ultrasound following exclusion of alcohol consumption, viral hepatitis and medications [17].

### Statistical analysis

Data were expressed as means (95% CI). The 95^th^ percentile of serum ALT in normal group was determined separately for men and women to define new gender-specific upper limit of normal ALT.

To perform sensitivity and specificity calculations, clinically characterized samples with normal livers were combined with those participants with HBV and NAFLD. The receiver operating characteristic (ROC) curve was plotted for each disease and gender. The sensitivity and specificity of ALT ULN currently utilized (named as previous ULN) and the new derived one were plotted on the ROC curves. The concordance statistics and 95% CI of ALT in detecting HBV and NAFLD was calculated using Delong's method. For all analyses, a *P* value of <0.05 was considered statistically significant. Statistical analyses were performed by PASW Statistics 18.0 software (SPSS Inc, Chicago, IL, USA) and MedCalc 10.0 software (Mariakerke, Belgium).

## Results

### Defining the new upper limit of normal ALT in population with normal liver

**Figure 2:**
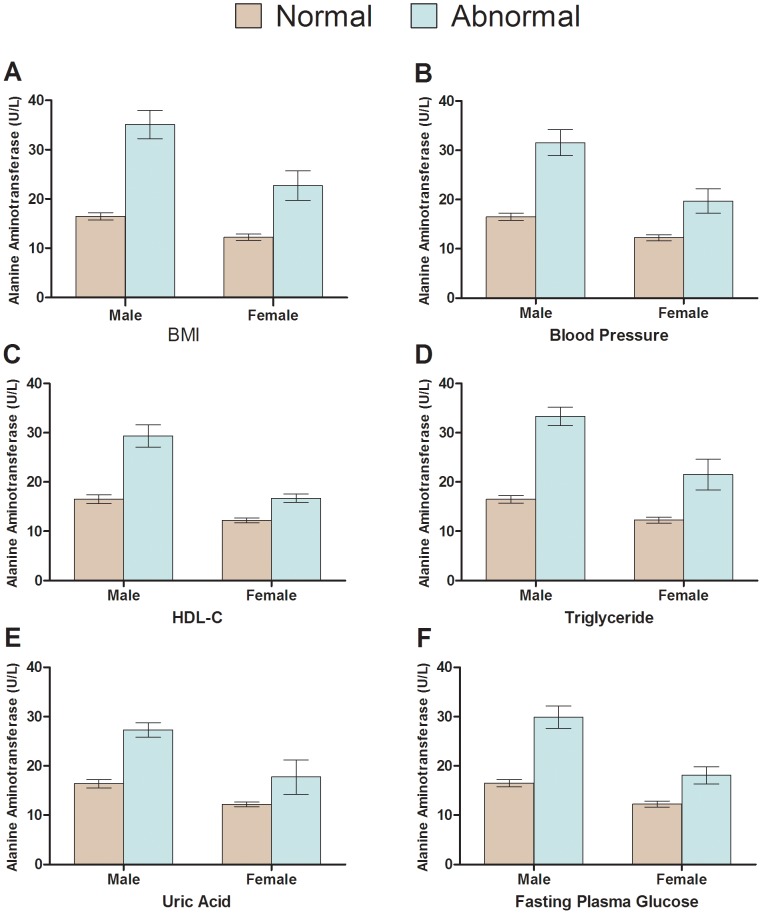
Comparing the effects of metabolic risk factors on alanine aminotransferase level. (A) body mass index (B) blood pressure (C) high-density lipoprotein cholesterol (D) triglyceride (E) uric acid (F) fasting plasma glucose. I bars indicate 95% confidence intervals.

In the current study, 53037 participants have been registered and the 95 percentile for ALT was 67.0 U/L in men and 46.6 U/L in women, which is compatible with the upper limit of normal ALT established in 1950s (ranging from 40 to 65 U/L). However in this population, there are several factors that may cause alternation of ALT. As shown in the Figure 1, after removing 512 participants due to missing ALT value, 15154 participants were excluded because of alcohol drinking. 3523 participants were found to be positive in viral infection such as HBV, HCV or HIV. 5598 participants were diagnosed as NAFLD according to the ultrasonography. 2198 participants had been in use of hepatotoxic medications or herbs, and 12415 participants had metabolic risk factors. Therefore, after excluding all these factors, the population with normal liver function remains at 13637 (4765 men and 8872 women) and was referred to the included sample in the Table 3 and Table S1. The 95 percentile of ALT in the population was 35.2 U/L and 23.4 U/L respectively. These values are considered as new upper limit of normal ALT in the current study. As Figure 2 had shown, each component of metabolic risk factors had significantly contributed to the increased alanine aminotransferase level.

### Comparison of the previous and new upper limit of normal ALT

#### Prevalence of abnormal ALT in China

**Figure 3:**
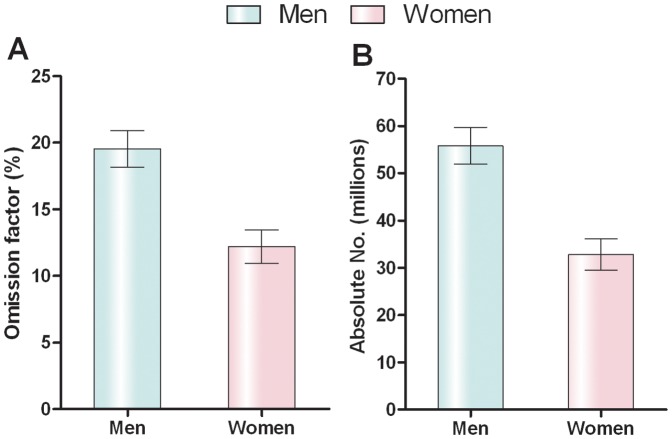
Omission factor (A) and absolute numbers (B) of cases among Chinese adults ages 19 to 44, according to men or women. I bars indicate 95% confidence intervals.

Since the new upper limit of normal ALT was lower than previous upper limit of normal ALT, the prevalence of abnormal ALT also changed according to the new ULN ALT value. By employing previous upper limit of normal ALT, 14.2% (95% CI: 13.7% to 14.7%) of men and 2.0% (95% CI: 1.8% to 2.2%) of women had abnormal ALT in the current health examination population (n = 53037). By using the new upper limit of normal ALT, 33.8% (95% CI: 33.1% to 34.5%) of men and 14.2% (95% CI: 13.7% to 14.8%) of women in this same population would have abnormal ALT. Therefore, there are 19.6% of men and 12.2% of women diagnosed as normal ALT according to the previous upper limit of normal ALT while they may be diagnosed as abnormal ALT according to the new upper limit of normal ALT. In the meanwhile, the proportions of HBV and NAFLD patients with elevated ALT increased significantly according to the new upper limit of normal ALT in genders (Table 4). Based on the census data of China in 2010 [18], this causes approximately 55,740,000 men and 32,820,000 women ages 19 to 44 years in China to be listed in different categories either as normal ALT according to the previous upper limit of normal ALT or as abnormal ALT according to the new upper limit of normal ALT (**Figure 3**).

**Table 4:** The proportions of HBV and NAFLD patients with elevated ALT according to their new upper limit of normal.

ALT ULN	HBV	NAFLD
Previous ULN		
Men	844/2278 (37.1%)	3025/5587 (54.1%)
Women	143/1245 (11.5%)	79/386 (20.5%)
New derived ULN		
Men	1055/2278 (46.3%)	3573/5587 (64.0%)
Women	444/1245 (35.7%)	209/386 (54.1%)

ALT, alanine aminotransferase; HBV, hepatitis B virus; NAFLD, nonalcoholic fatty liver disease; ULN, upper limit of normal.

### Sensitivity and specificity of the upper limit of normal ALT in detection of chronic liver disease

**Figure 4:**
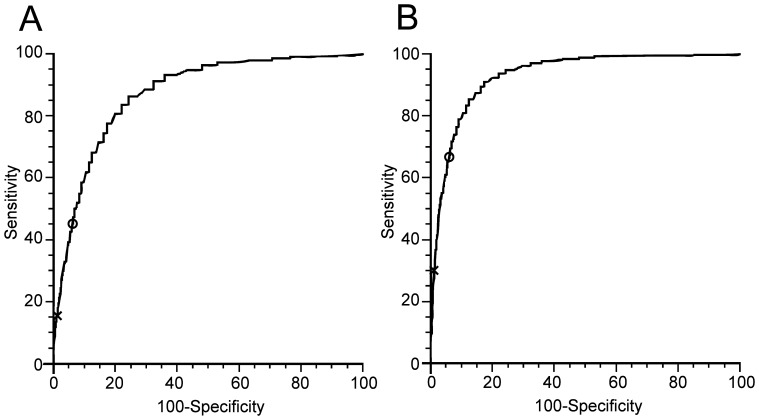
Receiver operating characteristic (ROC) curves for alanine aminotransferase (ALT) in men. Shown are the sensitivity and [100-specificity] of ALT in detecting hepatitis B virus infection (A) and nonalcoholic fatty liver disease (B). Plotted along the ROC curves are the ALT ULN currently used (marked with “x”) and the new derived ALT ULN (marked with “o”).

**Figure 5:**
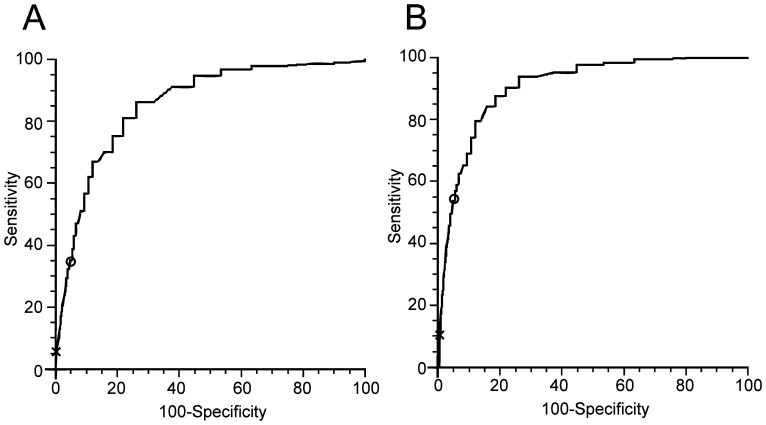
Receiver operating characteristic (ROC) curves for alanine aminotransferase (ALT) in women. Shown are the sensitivity and [100-specificity] of ALT in detecting hepatitis B virus infection (A) and nonalcoholic fatty liver disease (B). Plotted along the ROC curves are the ALT ULN currently used (marked with “x”) and the new derived ALT ULN (marked with “o”).

For examination of the upper limit of normal ALT in liver disease, we divided the population into three groups: normal liver group with 13637 participants, chronic HBV with 3523 participants and NAFLD with 5598 participants. By employing statistical analysis, ROC curves were developed separately for men and women (Figure 4 and 5), which indicate the sensitivity and specificity of the upper limit of normal ALT. As shown in the Figure 4, the concordance statistics for detection of liver disease were 0.873 (95%CI: 0.865–0.881) for HBV and 0.932 (95%CI: 0.927–0.937) for NAFLD in men while in women the concordance statistics for detection of liver disease were 0.857 (95%CI: 0.850–0.864) for HBV and 0.909 (95%CI: 0.903–0.915) for NAFLD (Figure 5).

According to ROC curves, the sensitivity and specificity of the new and previous upper limit of normal ALT were shown in Table 5. When the previous upper limit of normal ALT was used, the sensitivity was low for detection of HBV and NAFLD in men (15.8% for HBV and 29.7% for NAFLD) and women (6.6% for HBV and 10.9% for NAFLD). However, the sensitivity was greatly increased to 39.4% for HBV and 61.1% for NAFLD in men and 35.3% for HBV and 53.8% for NAFLD in women when the new upper limit of normal ALT value was used. Although the sensitivity of the new upper limit of normal ALT was increased, the specificity was decreased from 98.7% for men and 99.4% for women with the previous upper limit of normal ALT to 94.8 for men and 94.6 for women with the new upper limit of normal ALT respectively (Table 5).

**Table 5:** Sensitivity and specificity of alanine aminotransferase, stratified by gender.

ALT ULN	Sensitivity (95%CI)		Specificity (95%CI)
	HBV	NAFLD	
Previous ULN			
Men	15.84 (14.2–17.7)	29.68 (28.4–30.9)	98.68 (98.3–99.0)
Women	6.61 (5.3–8.2)	10.93 (7.9–14.6)	99.39 (99.2–99.5)
New derived ULN			
Men	39.35 (37.0–41.7)	61.09 (59.7–62.4)	94.84 (94.2–95.4)
Women	35.27 (32.6–38.0)	53.83 (48.6–59.0)	94.61 (94.1–95.1)

ALT, alanine aminotransferase; HBV, hepatitis B virus; NAFLD, nonalcoholic fatty liver disease; CI, confidence interval; ULN, upper limit of normal.

## Discussion

This analysis of a large population-based sample demonstrated that the statistically defined ALT threshold was about half of the currently used value for Chinese adults. The analysis of diagnostic performance in adults with and without CLD showed that the currently used ULN ALT value had low sensitivity for determining CLD in Chinese population. However, the gender-specific ALT threshold newly derived from the analyzed population doubled the sensitivity in men and fivefold the sensitivity in women with only a small reduction in specificity.

To our knowledge, it was the first time to re-evaluate the upper limit of normal ALT in China in past 20 years. Based on the results we had found, it would greatly increase the number of asymptomatic patients with abnormal ALT values–in China alone, by many millions of patients each year, and would identify more patients with NAFLD and clinically mild HBV infection. Many persons with potentially treatable liver diseases might be omitted or exposed to unnecessary risk by taking potentially hepatotoxic medicines.

We highlighted the importance of metabolic factors in determining ALT levels [19], [20]. Although the mildness of laboratory abnormalities rendered liver biopsy analyses unjustifiable, the noninvasive work-up of patients with ALT abnormalities of unknown origin indicated that more than 80% of such patients were overweight or had a hyperechoic hepatic pattern on ultrasonography. These findings were not unexpected; the prominent role of fat accumulation in inducing CLD has been previously assessed in the general population and in patients with overweight or obesity and those with diabetes or prediabetes [21], [22], [23].

We also found that exclusion of participants with NAFLD or HBV infection had a visible effect on distribution of ALT levels. In fact, for both genders, the 95^th^ percentiles of the crude distributions of ALT level were substantially lower than the normal limits adopted at our hospital over the past 25 years. We also observed that this reduction was more marked in women (from 55 IU/ml to 23 IU/ml, or 60%) than in men (from 55 IU/ml to 35 IU/ml, or 35%). We hypothesize that undiagnosed NAFLD or HBV infection had played an important role in previous overestimates of ALT thresholds for liver disease.

Nowadays, drug-induced liver injury is increasingly being recognized as a cause of clinically significant acute and chronic liver disease [24]. However, the lack of objective confirmatory diagnostic tests coupled with the highly variable clinical presentation could often lead to a delay in recognition even misdiagnosis. In this study, we comprehensively investigated 218 kinds of drugs (including 168 medications and 50 herbs) that are known to be associated with hepatotoxicity and we strictly excluded the participant that had taken one or more of these drugs 2 weeks before the enrollment.

In real world situation, we propose that persons who repeatedly demonstrate ALT abnormalities according to the updated healthy limits should be further investigated for liver disease by using serum biochemistry analyses, hepatitis virus tests, and hepatic ultrasonography. The cost-effectiveness and long-term effect of this approach on the natural course of liver disease need to be further assessed prospectively.

This study had potential weaknesses. First, ALT measurement was made at a single point. Thus, intra-individual variability in ALT with repeated measurement was not assessed. Furthermore, dynamic analysis of ALT was more important and meaningful than a single measurement. Second, it was a cross-sectional design; the study population came exclusively from a single center. Multicenter, long follow-up period studies of larger populations are needed. Third, the diagnosis of NAFLD was based on ultrasonographic examination, which was not sensitive enough to detect mild steatosis. However, this method was widely used in epidemiological studies of NAFLD because it was non-invasive, safe, widely available, portable and the sensitivity and specificity for detecting hepatic steatosis was acceptable. Proton-magnetic resonance spectroscopy would be used in our future work. Finally, histological assessment of disease severity was absent in current study.

In conclusion, currently used ALT ULN in adult is set too high to reliable detect CLD in China. The updated definition of normal adults ALT allows greater sensitivity in diagnosing early liver disease. By identifying those with HBV and NAFLD, targeted interventions can be implemented to minimize further health-related morbidity.

## Supporting Information

Figure S1 Biochemical and metabolic characteristics in three groups (normal liver, HBV and NAFLD). (A) Alanine aminotransferase, ^*^: *P* = 0.0001. (B) Body mass index, ^*^: *P* = 0.0001. (C) Uric acid, ^*^: *P* = 0.0001. (D) Blood pressure, ^*^: *P* = 0.0001. (E) Blood lipids and FPG, ^*^: *P* = 0.0001, ^**^: *P* = 0.035, ^***^: *P* = 0.018. I bars indicate 95% confidence intervals. HBV, hepatitis B virus; NAFLD, nonalcoholic fatty liver disease; SBP, systolic blood pressure; DBP, diastolic blood pressure; HDL-C, high-density lipoprotein cholesterol; LDL-C, low-density lipoprotein cholesterol; FPG, fasting plasma glucose; TC, total cholesterol; TG, triglyceride.
(JPG)

Table S1 **Demographic and Metabolic Characteristics of the Included and Excluded Sample.**
(DOC)
