# Supplementary figures and images for: Upper Limits of Normal for Serum Alanine Aminotransferase Levels in Chinese Han Population
Source: PLoS One. 2012 Sep 4;7(9):e43736. doi: 10.1371/journal.pone.0043736 (PMC3433469; doi:10.1371/journal.pone.0043736)

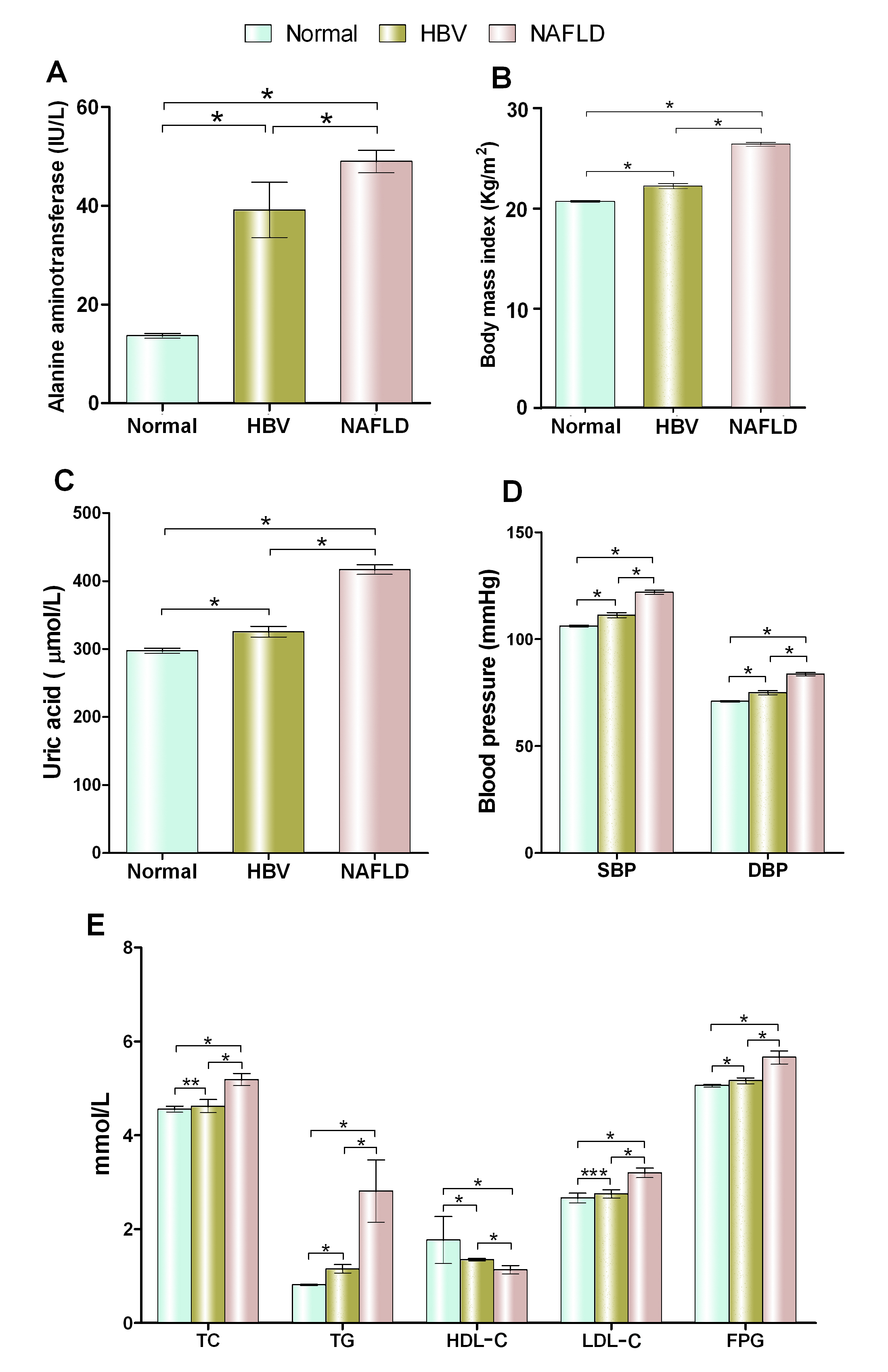

Supplement: Figure S1 — Biochemical and metabolic characteristics in three groups (normal liver, HBV and NAFLD). (A) Alanine aminotransferase, *: P = 0.0001. (B) Body mass index, *: P = 0.0001. (C) Uric acid, *: P = 0.0001. (D) Blood pressure, *: P = 0.0001. (E) Blood lipids and FPG, *: P = 0.0001, **: P = 0.035, ***: P = 0.018. I bars indicate 95% confidence intervals. HBV, hepatitis B virus; NAFLD, nonalcoholic fatty liver disease; SBP, systolic blood pressure; DBP, diastolic blood pressure; HDL-C, high-density lipoprotein cholesterol; LDL-C, low-density lipoprotein cholesterol; FPG, fasting plasma glucose; TC, total cholesterol; TG, triglyceride. (JPG) [file pone.0043736.s001.jpg]
