# Supplementary material for: Upper Limits of Normal for Serum Alanine Aminotransferase Levels in Chinese Han Population
Source: PLoS One. 2012 Sep 4;7(9):e43736. doi: 10.1371/journal.pone.0043736 (PMC3433469; doi:10.1371/journal.pone.0043736)
Supplement: Table S1 — Demographic and Metabolic Characteristics of the Included and Excluded Sample. (DOC) [file pone.0043736.s002.doc]

| **Table S1. Demographic and Metabolic Characteristics of the Included and Excluded Sample.** | | | | | |
| --- | --- | --- | --- | --- | --- |
|  | Included sample | |  | Excluded sample | |
| Characteristic | Men (n = 4765) | Women (n = 8872) |  | Men (n = 23583) | Women (n = 15305) |
| Mean age (95% CI)--(year) | 33.3 (33.1-33.4) | 34.4 (34.2-34.5) |  | 36.3 (36.2-36.4) | 35.7 (35.4-35.9) |
| Mean BMI (95% CI)-- (kg/m^2^) | 21.13 (21.08-21.19) | 20.39 (20.35-20.43) |  | 25.64 (25.57-25.71) | 22.27 (22.10-22.43) |
| Mean SBP (95% CI)-- (mmHg) | 109.8 (109.7-110.1) | 104.1 (103.9-104.3) |  | 120.7 (120.4-121.0) | 109.1 (108.4-109.7) |
| Mean DBP (95% CI)--(mmHg) | 73.4 (73.3-73.6) | 69.6 (69.4-69.7) |  | 82.7 (82.5-82.9) | 73.2 (72.7-73.6) |
| Mean total cholesterol (95% CI)-- (mmol/L) | 4.55 (4.51-4.60) | 4.56 (4.53-4.59) |  | 5.09 (5.05-5.14) | 4.62 (4.57-4.69) |
| Mean triglyceride (95% CI)-- (mmol/L) | 0.96 (0.95-0.97) | 0.73 (0.72-0.74) |  | 2.46 (2.31-2.67) | 1.11 (1.07-1.15) |
| Mean HDL-C (95% CI)-- (mmol/L) | 1.48 (1.39-1.59) | 1.89 (1.64-2.23) |  | 1.14 (1.13-1.18) | 1.44 (1.42-1.47) |
| Mean LDL-C (95% CI)-- (mmol/L) | 2.83 (2.77-2.92) | 2.57 (2.55-2.60) |  | 3.13 (3.10-3.16) | 2.72 (2.67-2.76) |
| Mean FPG (95% CI)-- (mmol/L) | 5.10 (5.08-5.11) | 5.04 (5.03-5.05) |  | 5.55 (5.52-5.59) | 5.27 (5.19-5.37) |
| Mean uric acid (95% CI)-- (µmol/L) | 360.6 (358.3-362.8) | 263.8 (262.6-265.1) |  | 410.9 (408.6-413.3) | 276.6 (273.6-280.0) |
| Mean ALT (95% CI)-- (IU/L) | 16.5 (16.1-16.8) | 12.2 (12.0-12.5) |  | 50.1 (49.0-51.1) | 29.6 (27.8-31.5) |
| ALT, alanine aminotransferase; BMI, body mass index; SBP, systolic blood pressure; DBP, systolic blood pressure; HDL-C, high-density lipoprotein cholesterol; LDL-C, low-density lipoprotein cholesterol; FPG, fasting plasma glucose; CI, confidence interval. | | | | | |
